# Start codon targeted (SCoT) polymorphism reveals genetic diversity in wild and domesticated populations of ramie (*Boehmeria nivea* L. Gaudich.), a premium textile fiber producing species

**DOI:** 10.1016/j.mgene.2015.01.003

**Published:** 2015-02-20

**Authors:** Pratik Satya, Maya Karan, Sourav Jana, Sabyasachi Mitra, Amit Sharma, P.G. Karmakar, D.P. Ray

**Affiliations:** a ICAR-Central Research Institute for Jute and Allied Fibres, Barrackpore, Kolkata 700120, India; b ICAR-National Institute of Research on Jute and Allied Fibre Technology, Kolkata 700040, India

**Keywords:** *Boehmeria nivea*, Ramie, SCoT, Genetic diversity, AMOVA, Population structure, India

## Abstract

Twenty-four start codon targeted (SCoT) markers were used to assess genetic diversity and population structure of indigenous, introduced and domesticated ramie (*Boehmeria nivea* L. Gaudich.). A total of 155 genotypes from five populations were investigated for SCoT polymorphism, which produced 136 amplicons with 87.5% polymorphism. Polymorphism information content and resolving power of the SCoT markers were 0.69 and 3.22, respectively. The Indian ramie populations exhibited high SCoT polymorphism (> 50%), high genetic differentiation (G_ST_ = 0.27) and moderate gene flow (N_m_ = 1.34). Analysis of molecular variance identified significant differences for genetic polymorphism among the populations explaining 13.1% of the total variation. The domesticated population exhibited higher genetic polymorphism and heterozygosity compared to natural populations. Cluster analysis supported population genetic analysis and suggested close association between introduced and domesticated genotypes. The present study shows effectiveness of employing SCoT markers in a cross pollinated heterozygous species like *Boehmeria*, and would be useful for further studies in population genetics, conservation genetics and cultivar improvement.

## Highlights

• Genetic diversity and population structure of ramie (*Boehmeria nivea*) were assessed by SCoT markers.
• SCoT markers revealed high polymorphism and resolving ability in *Boehmeria nivea*.
• The wild ramie showed moderate heterozygosity and gene flow with high genetic variation, suggesting unidirectional gene flow.
• The domesticated population exhibited highest heterozygosity and close association with introduced population.

## Introduction

Ramie or China grass (*Boehmeria nivea* L. Gaudich., Urticaceae) is an industrially important crop. The principal end product is textile grade fiber, which is considered to be the longest and strongest in the plant kingdom (Kozlowaski et al., 2005). Ramie fiber is fine, lustrous, durable and resistant to microbial degradation with high moisture absorbing capacity (Sarkar et al., 2010). Fresh ramie leaves and tops are used as animal feed and green manure (Brink, 2011). The leaf and the root extracts of the plant have antimicrobial (Brink, 2011, Xu et al., 2011), anti-inflammatory, antioxidant (Lin et al., 1998) and hepatoprotective (Huang et al., 2009) properties. As a high biomass producing crop, ramie also has high potential for phytoremediation (Yang et al., 2010).

The crop is cultivated in China, Brazil, Lao PDR, Philippines, India, South Korea and Thailand. Ramie has been grown in China and Indo-Malay peninsula for at least 5000 years (Kirby, 1963, Liang et al., 2009). In India, ramie is distributed in the North East regions, particularly in the states of Assam, Meghalaya and Arunachal Pradesh, which fall under the Indo-Malay center of origin of the species (Sarma, 2008). *B. nivea* in its natural habitat reproduces sexually through seed formation via open pollination and asexually through underground rhizomes. Despite a long history of natural occurrence and cultivation, no report is yet available on genetic variability and population structure of ramie from India. Genetic variability in a natural population is crucial for evolutionary fitness and ecological adaptation (Hughes et al., 2008, Poczai et al., 2012) while diversity in cultivated population is crucial for genetic improvement. In order to prevent the loss of *B. nivea* from its most important natural habitat in India and to effectively utilize the wild genetic resources in crop improvement it is very necessary to study the genetic diversity of this species.

Cultivar differentiation and genetic diversity assessment using morphological markers are challenging in a clonally propagated species, as the clones are expected to be heterozygous. Molecular markers having higher precision and resolvability are a better option for genetic differentiation, diversity studies and analysis of population structure. Genetic diversity in Chinese ramie cultivars has been estimated using RAPD, ISSR, SSR and SRAP markers (Guo et al., 2003, Liu et al., 2008, Liu et al., 2009, Zhou et al., 2004) but studies on the genetic structure of wild populations are limited. Of the various DNA marker systems, start codon targeted (SCoT) polymorphism (Collard and Mackill, 2009) is gaining popularity for its superiority over other dominant DNA marker systems like RAPD and ISSR for higher polymorphism and better marker resolvability (Gorji et al., 2011). The SCoT primers are based on conserved regions flanking the initiation codon sequences of genes. It shares the principle of using a single primer like RAPD and ISSR. The marker system has been successfully employed in genetic diversity analysis and fingerprinting of a number of agricultural and horticultural crop species (Luo et al., 2010, Mulpuri et al., 2013, Xiong et al., 2011) and in population structure analysis of mushrooms (Zhao et al., 2013). In the present study, we employed SCoT markers to i) examine the utility of SCoT marker for genetic analysis in ramie, ii) reveal the genetic structure of Indian ramie populations and iii) study the genetic relatedness of Indian and introduced ramie genotypes with domesticated cultivars. This is the first report on population structure analysis of Indian ramie and perhaps the only study on the use of SCoT markers for genetic analysis of outbreeding semi-perennial species.

## Materials and methods

### Plant material

**Fig. 1:**
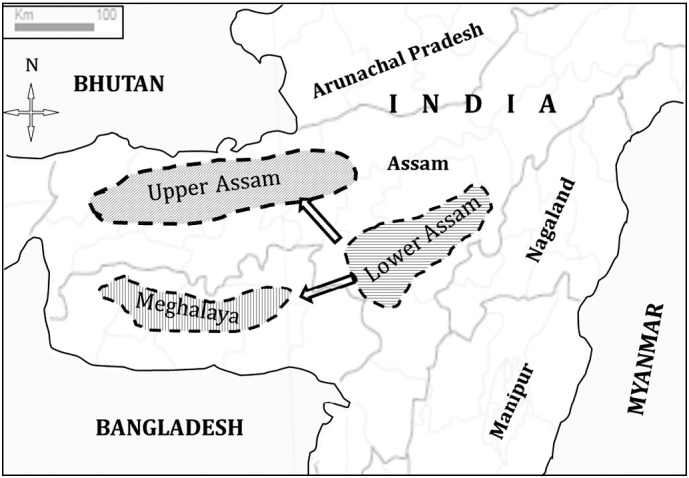
A representative outline of geographical distribution of Indian ramie genotypes sampled for the present study. The arrows indicate direction of gene flow among populations.

The experimental set consisted of 155 samples of *B. nivea* from three natural populations, introductions and breeding materials. A total of 82 genotypes representing three Indian populations and introduced population (17 genotypes) were selected for the present study. The Indian populations were collected from the Upper Assam region, Lower Assam region and Meghalaya (Fig. 1), which is considered the natural habitat of Indian ramie and a part of the Indo-Malay center of origin and diversity of ramie (Sarma, 2008). The exotic population was introduced in India during the 1960s to evaluate the potential of new introductions (Sarma, 2008). To assess the genetic relationship of the natural population and the domesticated improved populations of ramie, a set of domesticated population comprising of 56 genotypes were also included in the study.

### DNA extraction

Genomic DNA was extracted from young leaf tissue following the Cetyltrimethylammonium bromide (CTAB) based protocol of Murray and Thomson (1980) with minor modifications. Briefly, the fresh leaves were homogenized in 20 ml extraction buffer (2% CTAB, 1.4 M NaCl, 100 mM Tris, 20 mM EDTA and 1.5% β-mercaptoethanol) at room temperature using a mortar and pestle, and the extract was incubated at 65 °C for 1 h. DNA was isolated by chloroform: isoamylalcohol (24:1, v/v), treated with RNase A (100 μg/ml, 30 min. at 37 °C) to avoid RNA contamination, precipitated with isopropanol and washed with ethanol. The quality of the DNA was checked by a UV–vis spectrophotometer (Eppendorf, Germany) by checking the A_260_/A_280_ ratio. The final concentration of DNA was adjusted to 50 ng/μl. All the DNA samples were stored at − 20 °C for genotyping.

### PCR amplification and SCoT variability

A total of 20 SCoT primers developed by Collard and Mackill (2009) were selected for the present study (Table 1). For optimization, PCR amplification was tested with different concentrations of Taq polymerase (0.8–2 U), MgCl_2_ (1–3 mM) and dNTPs (0.1–0.4 mM). The final PCR cocktail (25 μl) contained 50 ng of template DNA, 1 × PCR buffer, 2.5 mM MgCl_2_, 0.2 mM dNTP_S_, 0.4 μM of primer and 1.5 U of Taq polymerase. The PCR programs were pre-run in a thermal cycler (BioRad, USA) at 94 °C for 4 min, followed by 43 cycles of 1 min at 94 °C, 1 min at annealing temperature (48–52 °C) and 2 min at 72 °C, with a final extension at 72 °C for 8 min. The PCR products were separated in 1.4% agarose gel in 1 × TAE buffer at 80 V for 1.5–2 h and visualized using a gel documentation system. To check the reliability of the amplification the experiments were repeated with random samples (30 no.).

### Data analysis

Reproducible, unambiguous SCoT amplicons were scored in a binary matrix as present (1) or absent (0) following Collard and Mackill (2009). Since it is a random multi-locus marker system like AFLP and RAPD, the polymorphic information content (PIC) value of the markers was calculated using the formula PIC_i_ = 2f_i_(1 − f_i_), where f_i_ is the frequency of amplified fragments at ith locus (De Riek et al., 2001, Mulpuri et al., 2013). The distinguishability of SCoT markers were determined by calculating resolving power (R_p_ = ΣI_b_), according to Prevost and Wilkinson (1999), where *I_b_* = 1 − (2 × |0.5 − *p*|); *p* being the proportion of genotypes containing a band. Mean resolving power ($\bar{Rp}$) was determined by dividing resolving power of a marker with the number of total bands amplified in that marker.

Genetic parameters were estimated as suggested for SCoT markers by Bhattacharyya et al. (2013). Estimation of diversity parameters like Percent polymorphism (*Pp*), number of observed (*N_a_*) and effective alleles (*N_e_*), Shanon's information index (*I*), total genetic diversity (*Ht*), population genetic diversity (*Hs*), Nei's expected heterozygosity (*h*), genetic identity (*I*) and genetic distance (*D*) were determined using POPGENE version 1.31 (Yeh et al., 1999). Estimate of gene flow (*Nm*) among the populations was obtained following *Nm* = 0.25 × (1 − *G_ST_*)/*G_ST_*. Analysis of molecular variance (AMOVA) was performed in GenAlEx v.6.1 (Peakall and Smouse, 2006). We separately compared introduced and indigenous populations as well as natural and domesticated populations of ramie. Pairwise genetic similarity was determined by calculating Jaccard's similarity coefficient with 1000 iterations and converted to a dendrogram by weighted neighbor-joining method implemented in DARwin 5 (Perrier and Jacquemoud-Collet, 2006).

## Results

### SCoT polymorphism

The 20 SCoT primers amplified a total of 136 amplicons with a range of 4 to 10 bands per primer, of which 119 (87.5%) were polymorphic. Percent polymorphism varied from 20% to 100%, with 3–10 polymorphic bands per primer. The primers S13 and S31 exhibited the highest number of polymorphic bands (8). Polymorphism information content ranged from 0.25 (S12) to 0.93 (S6) with an average of 0.69. Only two primers, S7 and S12 showed low polymorphism (PIC < 0.5). The resolving power of the primers ranged from 1.24 (S19) to 5.00 (S13) with a mean value of 3.22. Mean resolving power over loci varied from 0.29 (S12) to 0.75 (S16), with an average of 0.50.

### Population structure

We first examined the genetic structure of Indian ramie populations. The average observed number of alleles (*Na*), effective number of alleles (*Ne*), Shannon's information index (*I*) and Nei's genetic diversity (*h*) in the Indian ramie populations were 1.52, 1.35, 0.32 and 0.21, respectively (Table 2). The population from Upper Assam exhibited higher values of *I* (0.29) and *h* (0.21) than the other two populations. The population of Upper Assam exhibited lower genetic polymorphism (40.8%) than the other two populations. The average SCoT polymorphism in Indian population was over 50%. Total genetic diversity (*Ht*) and population diversity (*Hs*) were estimated to be 0.36 and 0.18, respectively. Relative genetic differentiation was high (*G_ST_* = 0.27), revealing high gene flow (*Nm* = 1.34) among the three populations. Results from AMOVA identified significant variability among populations (13.1%, *ΦPT* = 0.18, P < 0.001), along with 60.3% within-population genetic variability. Nei's unbiased genetic distance between the three populations varied from 0.076 (Upper Assam and Lower Assam) to 0.099 (Upper Assam and Meghalaya) (Table 3).

The *Na*, *Ne*, *I* and *h* values of introduced ramie genotypes were observed as 1.84, 1.46, 0.43 and 0.28, respectively. The percentage of SCoT polymorphism was higher in domesticated population (88.5%) than in natural ramie populations (Table 2). The *Na* (1.94) and *Ne* (1.44) values for the domesticated population were high, being similar to that of introduced population. The group exhibited high percentage of polymorphic loci (95.4%) and heterozygosity (*h* = 0.42). The domesticated populations were genetically closer to introduced population, with a genetic identity of 0.953. Overall AMOVA revealed significant differences between the three groups (Indian, introduced and domesticated population) (*ΦPT* = 0.27, P < 0.001) (Table 4). The genetic variation among the groups accounted for 26.6% of the total variation.

### Cluster analysis

**Fig. 2:**
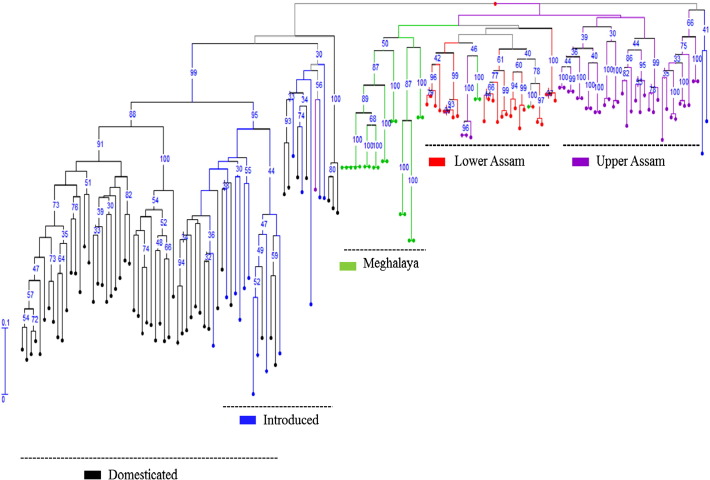
Dendrogram showing relationship of *B. nivea* populations based on Jaccard's similarity coefficient. Bootstrap values are indicated at each node.

The weighted neighbor joining based cluster clearly distinguished the Indian populations from the introduced ramie populations, showing distinct sub-clusters of the populations from the Upper Assam, Lower Assam and Meghalaya (Fig. 2). The population from Upper Assam exhibited two sub clusters. Two sub clusters were also identified in the ramie population of lower Assam. The Jaccard's similarity coefficient exhibited a mean value of 0.57 with a range of 0.00 to 0.86. The domesticated populations were grouped with the introductions, but not with natural populations of India.

## Discussion

### SCoT markers exhibit high polymorphism and resolving power

The SCoT markers are expected to be linked to functional genes and corresponding traits, thus the amplicons can be converted to gene targeted marker systems (Xiong et al., 2011). Besides these markers are multilocus, which are helpful in obtaining high genetic polymorphism. The number of amplicons and PIC of the SCoT markers observed in ramie from the present study is comparable to the results obtained in other studies such as in groundnut (Xiong et al., 2011), mango (Luo et al., 2010), and *Dendrobium nobile* (Bhattacharyya et al., 2013). The resolving power of SCoT markers in ramie was higher than in potato (Gorji et al., 2011) and similar to resolving power of SCoT markers in *Dendrobium*. Since ramie is a cross-pollinated species, genetic variability is expected to be higher in ramie than in potato, which is a self-pollinated crop. *Dendrobium*, on the other hand might have a more complex genetic structure due to self-incompatibility and chimeric polyploidy (Kuehnle, 2007). Information on the resolving power of other marker systems in ramie is not available, although like SCoT, high polymorphism (> 90%) was also reported for RAPD and ISSR (Liu et al., 2009), whereas SRAP markers were marginally less polymorphic (85.5%) (Liu et al., 2008). High polymorphism and resolving power of SCoT markers in ramie would be more useful for DNA fingerprinting, population structure analysis and effective management of genetic resources in ramie.

### SCoT markers can be used to study breeding history

The genetic polymorphism generated by SCoT markers can also be used for tracing and reconstruction of breeding history of the domesticated genotypes. The introduced ramie accessions exhibited close genetic association with the domesticated genotypes, but not with the Indian natural ramie populations. Our results show that initiation of ramie breeding in India relied primarily on the introduction of new cultivars from other countries. The Indian native ramie has higher adaptability; thus this gene pool is a valuable reservoir for enriching genetic improvement programs targeting higher yield, better quality, adaptability and resistance to pests and diseases.

### Population structure of Indian ramie indicates high vulnerability and unidirectional gene flow

Assessment of genetic diversity is a prerequisite for efficient genetic resource management, conservation of species in natural habitat and identification of suitable parental combinations, all of which produce perceptible impacts on genetic improvement of a crop species (Poczai et al., 2012). Little information is available on genetic structure of semi-perennial species with multiple reproduction mechanisms. Ramie is principally propagated by rhizome in cultivated populations (Sarkar and Maitra, 2005), but a complex outcrossing mechanism exists to produce viable seeds (Xing et al., 2009). The sexual propagation contributes to the genetic variability in wild populations but not in the cultivated population maintained by clonal propagation. Ramie exhibits complex outcrossing behavior including monoeceae and occasional gynodioeceae depending on the environment and habitat (Liu and Zhou, 2005). Generally, wind pollinated outcross species exhibit higher within-population genetic diversity with low level of genetic differentiation and gene flow among populations (Hamrick and Godt, 1996, Torres-Díaz et al., 2007). While analyzing population structure of ramie, a wind pollinated species, we found higher within-population diversity than diversity among populations, meeting our expectations. High within population diversity indicates that these populations should be conserved in their natural habitat for preserving genetic diversity (Manners et al., 2013). However, G_ST_ and gene flow were moderate to high among these populations. Endemic perennials often exhibit high levels of gene flow (Hamrick and Godt, 1996). The high gene flow in these populations thus might have originated from endemic perennial nature of the species, or from the high amount of pollen travel due to small pollen size and high pollen load of individual plants. Close genetic association between ramie populations of lower Assam with both Meghalaya and Upper Assam indicates a gradient of pollen and gene flow from Meghalaya to Lower Assam and Upper Assam (Fig. 1). In contrast, the genetic distance between ramie populations of Upper Assam and Meghalaya are higher, which indicates a barrier of gene flow. The barrier might be attributed to the hilly terrains of the Meghalayan region, separating it from the plains of the Upper Assam region. The extent of heterozygosity in wild populations was found to be low. The native gene pool is poorly represented in the domesticated population. The area of adaptation of ramie is often roadsides and margins of forests, which are highly vulnerable to deforestation, modernization and urbanization. Thus conservation of the native ramie gene pool is absolutely essential to revive and maintain genetic variability in natural conditions.

### Domesticated population retains high heterozygosity

The domesticated genotypes exhibited high heterozygosity, which may have accumulated due to clonal propagation and perpetuation of somatic mutations. Higher heterozygosity and decreased population differentiation are characteristic of clonality, while the opposite is true for sexual propagation (Balloux et al., 2003). They also suggest that excess heterozygosity is a reliable indicator for identification of clonally propagated species, which is supported by the high heterozygosity observed in the domesticated ramie population. Such populations accumulate more heterozygosity through independent somatic mutations for several generations. Partial asexual reproduction also reduces allele segregation and in turn increases heterozygosity (Birky, 1996), which may explain the higher heterozygosity of the domesticated population.

## Conclusion

The present work is the first report on genetic variability of Indian ramie suggesting that the preservation of genetic variability in the natural populations should be given priority. Our work also establishes the usefulness of the SCoT marker system in genetic diversity studies, ancestry determination and population structure analysis of *B. nivea*. The findings will be useful for devising conservation strategies and for selecting a suitable gene pool for genetic improvement of this industrially important crop species.

## Figures and Tables

**Table 1:** Genetic polymorphism generated by 20 SCoT markers in ramie.

Sl no.	Primer name	Primer sequence (5′–3′)	NTB	NPB	PIC	Rp	$\bar{Rp}$
1.	S1	CAACAATGGCTACCACCA	5	3	0.50	2.84	0.57
2.	S2	CAACAATGGCTACCACCC	8	6	0.69	3.75	0.47
3.	S3	CAACAATGGCTACCACCG	5	4	0.82	2.38	0.58
4.	S6	CAACAATGGTCACCACGC	5	5	0.93	1.97	0.39
5.	S7	CAACAATGGTCACCACGG	10	5	0.46	3.81	0.38
6.	S11	AAGCAATGGCTACCACCA	5	3	0.63	2.80	0.56
7.	S12	ACGACATGGCGACCAACG	5	1	0.25	1.43	0.29
8.	S13	ACGACATGGCGACCATCG	10	8	0.71	5.00	0.50
9.	S14	ACGACATGGCGACCACGC	7	5	0.70	3.68	0.61
10.	S15	ACGACATGGCGACCGCGA	6	6	0.84	3.97	0.66
11.	S16	ACCATGGCTACCACCGAC	6	5	0.85	3.79	0.75
12.	S17	ACCATGGCTACCACCGAG	4	3	0.58	1.85	0.46
13.	S18	ACCATGGCTACCACCGCC	10	9	0.75	4.75	0.47
14.	S19	ACCATGGCTACCACCGGC	4	4	0.89	1.24	0.31
15.	S21	ACGACATGGCGACCCACA	9	6	0.59	3.64	0.45
16.	S22	AACCATGGCTACCACCAC	10	7	0.70	4.81	0.48
17.	S26	ACCATGGCTACCACCGTC	4	3	0.76	1.85	0.62
18.	S28	CCATGGCTACCACCGCCA	8	7	0.69	4.18	0.52
19.	S29	CCATGGCTACCACCGGCC	6	4	0.64	2.29	0.38
20.	S31	CCATGGCTACCACCGCCT	9	8	0.78	4.29	0.54

NTB, total number of bands amplified; NPB, number of polymorphic bands; PP, polymorphism (%), Rp, resolving power; $\bar{Rp}$, mean resolving power; J, Jaccard's similarity coefficient.

**Table 2:** Genetic diversity parameters for the ramie populations under study.

Population/group	Sample size	*Na* ± *SD*	*Ne* ± *SD*	*I* ± *SD*	*h* ± *SD*	*Pp*	*Ht*	*Hs*	*G_ST_*	*Nm*
Lower Assam	24	1.12 ± 0.07	1.22 ± 0.03	0.21 ± 0.02	0.14 ± 0.01	40.8				
Upper Assam	36	1.42 ± 0.07	1.35 ± 0.04	0.29 ± 0.03	0.21 ± 0.04	52.3				
Meghalaya	22	1.27 ± 0.07	1.30 ± 0.03	0.28 ± 0.02	0.18 ± 0.02	58.5				
Introduced	17	1.84 ± 0.04	1.46 ± 0.03	0.43 ± 0.02	0.28 ± 0.01	88.5				
Domesticated population	56	1.94 ± 0.03	1.44 ± 0.03	0.42 ± 0.01	0.27 ± 0.01	95.4				
Total	155	1.52 ± 0.03	1.35 ± 0.01	0.32 ± 0.01	0.21 ± 0.01	59.87 ± 9.04	0.36	0.18	0.52	0.46

**Table 3:** Nei's unbiased genetic distance among the natural and breeding populations of ramie.

	Upper Assam	Lower Assam	Meghalaya	Domesticated population
Introduced	0.205	0.236	0.195	0.047
Upper Assam		0.076	0.099	0.266
Lower Assam			0.077	0.289
Meghalaya				0.248

**Table 4:** Variability in ramie populations under study revealed by AMOVA.

Source	df	Mean square	Estimated variation	Variation (%)	*ΦPT*	P value
Among populations	4	103.80	3.32	13.1	0.18	< 0.001
Among groups	2	424.80	6.76	26.6	0.27	< 0.001
Within populations	148	16.35	15.33	60.3	0.40	< 0.001
